# The Effect of Unilateral and Bilateral Leg Press Training on Lower Body Strength and Power and Athletic Performance in Adolescent Rugby Players

**DOI:** 10.5114/jhk/159626

**Published:** 2023-01-20

**Authors:** Xiang Zhao, Anthony P. Turner, John Sproule, Shaun M. Phillips

**Affiliations:** 1 Institute of Sport, Physical Education and Health Science, University of Edinburgh, Edinburgh, Scotland, UK

**Keywords:** single-leg, bilateral deficit, linear sprint, vertical jump

## Abstract

This study aimed to compare the effects of 5 weeks of unilateral and bilateral leg press training on lower body strength, linear sprinting and vertical jumping performance in adolescent rugby players. Twenty-six male adolescent rugby players (age = 15.3 ± 0.4 years) were assigned via stratified block randomization to unilateral (n = 9), bilateral (n = 9) and control (n = 8) groups. Training consisted of either the unilateral or the bilateral leg press twice weekly over five weeks, with the control group maintaining habitual training. Lower body unilateral and bilateral strength, vertical jump and linear sprint performance were assessed before and after training. After 5 weeks of training, both training groups significantly increased the 5-repetition maximum bilateral leg press (unilateral group = 8.9%, d = 0.53; bilateral group = 10.9%, d = 0.55, p < 0.01) and the 5-repetition maximum unilateral leg press (unilateral group = 20.2%, d = 0.81; bilateral group = 12.4%, d = 0.45, p < 0.01). There was no significant difference between the size of improvement in unilateral and bilateral groups in the 5-repetition maximum bilateral leg press, but the 5-repetition maximum unilateral leg press increased significantly more in the unilateral group (p < 0.05). No significant training effects were found for vertical jump or linear sprint performance. The results indicated that unilateral leg press training was as effective as bilateral leg press training in improving bilateral strength and more effective in improving unilateral strength in adolescent rugby players. However, strength improvement did not transfer to athletic performance improvements in either group

## Introduction

Rugby is a sport demanding a high level of physical fitness, with players performing frequent bouts of intense activity involving sprinting, jumping, physical collision and tackles. Success in rugby is heavily reliant on players possessing appropriate levels of strength, power, speed, agility and endurance. With regard to muscular strength and power, there is evidence suggesting that elite rugby union players are generally stronger and more powerful than sub-elite players (Quarrie et al., 1995). Baker et al. (2008) also reported greater muscular strength and power measurements in elite junior rugby league players compared with sub-elite players. Well-developed muscular strength and power contribute to tackling ability, sprinting performance and injury prevention. Potential benefits of resistance training (RT) in youth rugby players include increased muscle strength, lower rates of sports-related injury, an increased bone strength index and an associated decreased risk of fractures (Faigenbaum et al., 2009; Myers et al., 2017). Evidence shows that regular participation in a RT program in a safe environment with correct exercise technique and proper supervision is safe for youth populations (Faigenbaum et al., 2010).

Traditionally, bilateral (BL) RT is more frequently implemented than unilateral (UL) RT, which tends to be used more as assistance training (McCurdy et al., 2005). Some authors have argued that BL RT is superior to UL as it allows greater absolute force production (Bobbert et al., 2006).

However, this argument can be challenged according to the ‘bilateral deficit’ phenomenon, where the maximal voluntary strength of both limbs contracting simultaneously is less than the sum of the maximal voluntary strength of each limb contracting independently, resulting in a higher relative intensity of work to be performed in UL than BL RT (Costa et al., 2015; Jones et al., 2012). In addition, many sporting movements are performed unilaterally or with weight transferred to one leg at a time, such as running, jumping, kicking and changing direction. According to the principle of specificity, training exercises closer to the specific exercise task are more likely to result in greater training outcomes. Additionally, in BL RT, the dominant limb may compensate for the weaker side, leading to uneven training loads, and the resulting imbalance may be a precursor to the potential musculoskeletal injury; by comparison, UL RT allows direct loads aimed for each limb, such that the between-limb asymmetries may be corrected to reduce the corresponding muscle imbalance.

Previous studies have demonstrated that BL and UL RT tends to improve muscle strength and power to a similar extent in healthy adults and adult rugby players (McCurdy et al., 2005; Speirs et al., 2016). However, these studies focused on comparing the effects of UL and BL resistance exercise using the back squat in an adult population (McCurdy et al., 2005; Speirs et al., 2016). It is widely considered that free weight RT promotes superior transfer to sports specific and motor performance compared to machine-based exercise and this has also been reported for the leg press (LP) (Wirth et al., 2016). In contrast to the back squat, the leg press is a closed kinetic-chain machine exercise widely used in athletic training and rehabilitation to enhance performance and promote functional movement patterns. One argument for the use of the LP in such settings is that compared with free weight lower body RT, the LP performed on a fixed trajectory with safety pins employed to limit the range of motion may be safer, particularly for UL activities. The LP overloads the gluteus maximus, hamstrings and vastus medial obliquus muscles and, moreover, has biomechanical and neuromuscular resemblance to many movements that are required during rugby, like running, sprinting and jumping. Previous studies have shown improved muscle strength, balance, vertical jump height, horizontal jump distance and sprinting ability in young and old adults (Correa et al., 2012; Pamukoff et al., 2014; Wawrzyniak et al., 1996) following LP training. Furthermore, the LP has been reported to improve 1- repetition maximum (RM) box squat strength by 50.4% after 15 weeks of training in adolescent rugby union players (Smart et al., 2013).

UL and BL squats can improve lower body strength and athletic performance to a similar extent in adults (Jones et al., 2012; Speirs et al., 2016; McCurdy et al., 2005). However, a comparison of UL and BL lower body strength training has not been carried out in youth athletes. Considering the benefit of the LP as a safe and effective exercise for improving muscular strength and athletic performance in adolescents, the purpose of this study was to compare the effectiveness of BL LP and UL LP training in lower body muscle strength, linear sprinting and vertical jumping performance in adolescent rugby players. We hypothesized that the UL LP and the BL LP would be equally effective in improving young adolescent rugby players’ lower body muscle strength, linear sprinting and vertical jumping performance, compared to a control group not undertaking LP training

## Methods

### Participants

Sample size estimation for the study was calculated from the equation recommended by Hopkins (2004). In this equation, CV refers to the coefficient of variation, and SWC refers to the smallest worthwhile change. The SWC was calculated as a factor of 0.2 of the between-subject standard deviation. According to the data collected from an initial pilot study and the published data (Dobbin et al., 2018), the calculated sample size was initially eight participants per group.


$$Sample\;size\;=\;8\;*\;\left(CV2/SWC2\right)$$

Twenty-six adolescent male school rugby union players (age = 15.3 ± 0.4 years, body height = 179.5 ± 6.5 cm, body mass = 74.5 ± 6.4 kg, maturity offset: +1.9 ± 0.4 years) participated in this study. Participants’ characteristics of all groups are presented in Table 1. A Fitquest Junior Children’s Physical Activity Readiness Questionnaire evaluated suitability to participate in the study. Inclusion criteria were healthy, no contraindications to RT, free of musculoskeletal injury and able to complete at least 10 repetitions of 36 kg (the minimum resistance of the LP machine) using the non-dominant leg. All participants regularly took part in school-based rugby training (2 × 60 min/week), a rugby match (1 × 60 min/week), RT (including traditional strength training and power training, 1 × 60 min/week) and physical education classes (2 × 50 min/week). Participants were requested not to partake in any additional lower body RT and the control group did upper body RT during the period of the study. All participants and their parents/guardians provided voluntary written informed consent after reading the information sheets. The study was approved by the Institutional Research Ethics Committee and conducted according to the Declaration of Helsinki.

**Table 1:** Descriptive statistics for anthropometrics per group (Mean ± SD).

Group	Number	Age (year)		Body height (cm)	Body mass (kg)	Maturity offset (year)
CON	8	15.3 ± 0.4	179.4 ± 5.2		75.2 ± 5.7	1.9 ± 0.4
UL	9	15.3 ± 0.3	178.9 ± 8.2		74.3 ± 7.7	1.9 ± 0.5
BL	9	15.2 ± 0.5	180 ± 4.5		74.2 ± 6.5	1.9 ± 0.4

BL = bilateral training group; UL = unilateral training group; CON = control group

### Design and Procedures

This study employed a stratified block randomized parallel pre-post measures design. Participants were randomly allocated to one of the training groups (UL and BL) or a control group (CON), with the blocking factor being maturity offset, expressed as age at peak height velocity (Mirwald et al., 2002). All groups maintained their habitual rugby training with the training groups receiving either UL or BL LP training twice weekly for five weeks with the same relative loads, while the CON did no additional training. Five-RM UL and BL LP, countermovement jump (CMJ), and 30-m linear sprint tests were conducted the week before and after training.

**Figure 1:**
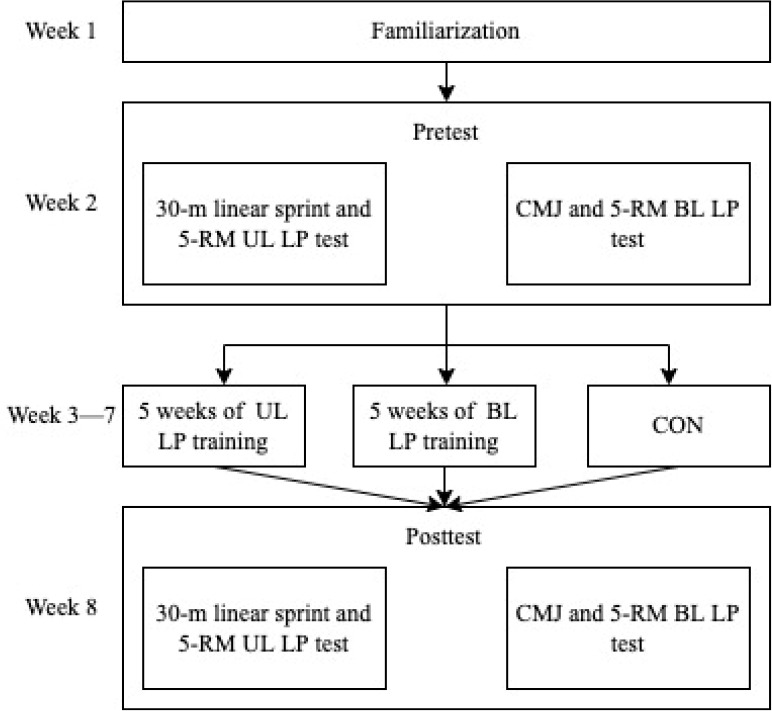
Study procedures. UL LP = unilateral leg press; BL LP = bilateral leg press; CON = control; CMJ = countermovement jump; RM = repetition maximum

The study procedure is shown in Figure 1. A 10-min standardized warm-up was performed at the beginning of all sessions consisting of five min jogging at approximately 10 km/hour, followed by a series of dynamic stretches with the emphasis on specific lower body movements, joint mobility and injury reduction. Dynamic stretches consisted of five movements (hand walk, lunge walk, high knee skip, heel ups, high knee run). Participants performed each dynamic stretch for a distance of 13 m, rested for 10 s at the end, then repeated the stretch for further 13 m. Verbal instructions and physical demonstrations were used to help participants maintain proper technique during all dynamic stretching exercises.

### Familiarization

In the first familiarization session, anthropometric data (body mass, body height, sitting height) were collected. The dominant leg, defined as the preferred leg for kicking a ball, was selected and used for UL LP exercises, tests and analysis. The UL and BL LP exercise techniques were explained verbally and demonstrated by the researcher. Each participant was guided to practice three sets of 10 repetitions of the UL and BL LP with 36 kg starting resistance and three min of rest intervals between each set. All participants were deemed able to perform the exercise with proper technique by the researcher and their coach. Following the practice, the predicted 1-RM UL and BL LP were measured. With assistance from the coach, participants were asked to choose a load that they believed they could lift five times. They were then asked to complete as many repetitions as possible at that load until they could not continue with the correct technique or the full range of motion. If the number of repetitions exceeded 10, another test was required after participants had fully recovered. The adjusted weight was based on how comfortably the participant performed the exercise during the last set. The purpose of measuring the predicted 1-RM strength test was to standardize the loads for familiarization and 5-RM strength measurement. All predicted 1-RM measures were determined within two trials. Predicted 1-RM was calculated using the following formula (Epley, 1985):


$$Predicted\;1-RM\;=\;\left(1+0.033\times repetitions\right)\;\times \;repetition\;weight$$

Two additional familiarization sessions were carried out, each separated by 48 hours. In familiarization session two, participants performed three sets of 10 repetitions at a sub-maximum (50% predicted 1-RM) load for the UL LP and the BL LP interspersed with three min recovery. Familiarization session three was similar to the second one with the load increased to 85% predicted 1-RM for five repetitions.

### Measures

#### Test Session 1

##### 30-m Linear Sprint

The 30-m linear sprint was evaluated two days after the last familiarization session in an indoor basketball hall with wooden floor by application of a high-speed smartphone camera and the MySprint app. The app measured time of the 40-m sprint showed perfect correlation with the photocell measurement (r = 0.989–0.999, standard error of estimate = 0.007–0.015 s) (Romero-Franco et al., 2017). With the frame by frame playback, this method allows for precise determination of the time when the participant crosses the marker. Using the recommended protocol detailed in Romero-Franco et al. (2017), the smartphone was set perpendicular to the track at the 15 m marker and at 10 m from the track and was mounted to a tripod at a height of 80 cm for smooth recording. A crash pad was placed 5 m from the end of the track to ensure participant’s safety. Three min following the standardized warm-up, participants performed three sprints separated by at least two min of passive recovery. Participants were instructed to use a crouching start position (staggered-stance) with their right hand on the start line of the track. They were asked to exert maximal effort and not to slow down until they reached a cone placed 2 m beyond the 30-m line. The best performance achieved in the three trials was taken as the representative value of this test.

### 5-RM UL LP Test

**Figure 2:**
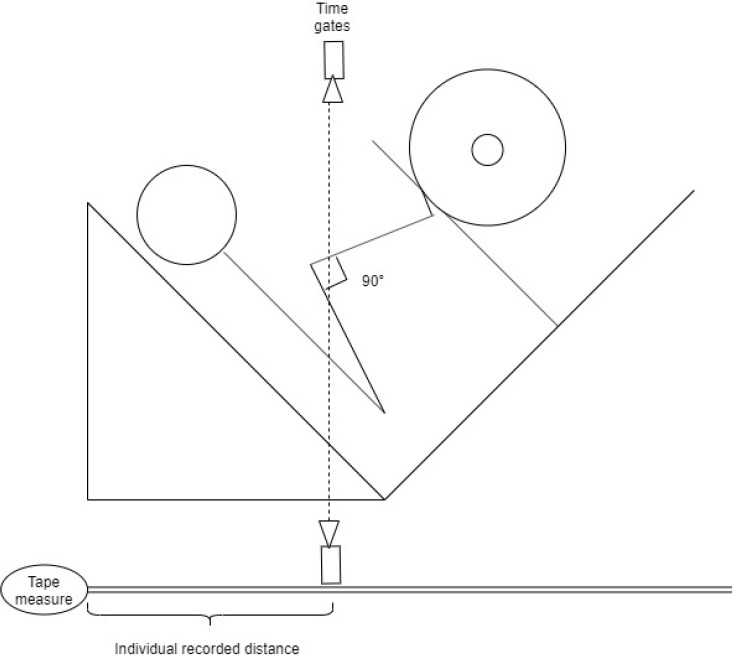
Individual recorded distance where the knee angle reaches 90°.

Five min after the 30-m linear sprint test, the 5-RM UL LP test took place. During all 5-RM tests, each participant was supervised by the same researcher who monitored the technique and the range of motion. The 5-RM test used the protocol adapted from Haff et al. (2015). Tape was placed 10 cm from the top of the LP machine footplate to standardize the participant’s foot placement. A set of timing gates (Brower Timing Systems, Utah, USA) were used to ensure participants reached the designated depth in order to standardize the movements. In UL and BL LP exercises, timing gates were set at the height of 50 cm. By moving the timing gates horizontally, participants could reach the designated 90° knee joint angle, determined by a right-angled object with the beam from the timing gates as shown in Figure 2.

Participants sat on the linear LP machine (Hammer strength 45° Plate-Loaded linear Leg Press; Illinois, United States) with their lower back, hips, and buttocks pressed against the pad, their foot placed at the center of the footplate marked with the tape and their hands holding the handles on the side of the machine. Participants attempted five repetitions starting from 50% of the estimated 1-RM measured during the familiarization session. All participants were requested to exert maximal effort during the tests. A successful lift was performed when the beam of the timing gate was broken at the end of the eccentric phase. At this point, participants were required to start the concentric contraction immediately rather than going beyond the required depth. If participants achieved five repetitions, the load was increased by 10–20% after three min of passive recovery until a successful 5-RM set could not be completed with a full range of motion; then, the load was reduced by 5–10% until five repetitions were completed. If participants deviated in any way from the required technique, the attempt was voided. All tests were completed within five attempts, thereby minimizing the potential confounder of cumulative fatigue. Two pieces of cardboard were used to shield the load on the LP machine from the vision of the participant. For this test, our unpublished data showed a high level of test-retest reliability in a similar participants’ group (adolescent school rugby players) with an intra-class correlation (ICC) = 0.97, coefficient of variation (CV) = 4.6% and smallest worthwhile change (SWC) = 5.8 kg.

### Test Session 2

#### Countermovement Jump Test

Two days after the first testing session, test session two took place. Following completion of the standardized warm-up, participants performed the CMJ test with hands placed on the hips to eliminate arm assistance while the feet were shoulder-width apart. Participants were instructed to stand still on the force plate (Kistler K2875A, Kistler, Winterhur Switzerland) for 5 s, then rapidly squat down to a self-selected depth and immediately perform an explosive CMJ upright with legs extended. Following the jump, participants were instructed to land back on the force plate and stand still until the completion of data collection. Three jumps were performed with a one-min rest interval between each attempt. The highest CMJ was recorded for further analysis. *5-RM BL LP Test*

Five min after the CMJ test, the 5-RM BL LP test was completed, similar to the UL LP test. Participants sat on the LP machine, feet were placed at the marked place on the footplate, and a successful press was performed when the knees reached 90° of flexion (end of the eccentric phase) before extension to the straight leg. For this test, our unpublished data showed a high level of test-retest reliability in a similar participants’ group (adolescent school rugby players) with an ICC = 0.98, CV = 7.6% and SWC = 10.4 kg.

### Training Intervention

Training was conducted twice weekly on two non-consecutive days in the morning for 5 weeks. The training period was selected in accordance with previous research that demonstrated positive adaptations in strength and athletic performance following short-term RT in adolescents (González-García et al., 2017; Gorostiaga et al., 1999) and to allow for two weeks of pre and post intervention tests within an education term. One-RM UL and BL LP loads were estimated based on individual 5-RM tests results. To be included in the final analyses, participants were required to complete at least 80% of the training sessions. Each training session consisted of the UL or BL LP with 7 repetitions at 70% 1RM, 4 repetitions at 80% 1RM, 3 repetitions at 85% 1RM, and 2 repetitions at 90% 1RM with two min rest intervals between each set (Chelly et al., 2009). The aim of this RT program was to increase muscle strength as detailed by Blimkie et al. (1998). Participants were required to perform the exercise with a 2:0:1 tempo whereby the concentric and eccentric phases were completed in 1 and 2 s, respectively, with no pause between phases. A metronome was used to facilitate the completion of the exercises at an appropriate tempo. The intensity was standardized across groups using the same relative intensity at a percentage of 1-RM in each exercise and the UL group trained both legs individually. Three min of rest were allocated between sets. Verbal encouragement was given when participants struggled to move the weight. In the third week, individual estimated 1 RM (UL or BL LP) was reassessed using the same 5-RM strength test protocol and absolute training loads were adjusted accordingly to maintain progression. Training groups performed the UL or BL LP as the only lower body exercise, with no additional lower body strength training performed outside the intervention. Participants trained the UL LP on both legs, with the dominant leg used for UL strength statistical analysis.

### Statistical Analyses

All data are expressed as mean ± standard deviation (SD). Statistical analyses were performed using the Statistical Package for Social Sciences (SPSS) 22.0. Before using parametric tests, the assumption of normality was verified by the Shapiro-Wilk test. A two-factor mixed analysis of variance (ANOVA) (three conditions × two time points) was conducted, followed by paired t-tests as post hoc tests to compare the effects of training within each group. The Mauchly’s test was used to evaluate the homogeneity of variances. Effect sizes (ES) were also calculated using Cohen’s d, where appropriate, to provide the magnitude of treatment effects with 0.2, 0.5 and 0.8 considered to represent small, medium and large effects. A oneway ANOVA was also applied to compare group differences of anthropometric characteristics and group pre-to-post-test change scores in the event of a significant interaction effect of time × group with the Tukey’s post-hoc test used when appropriate. The criterion level for significance was set at p ≤ 0.05.

## Results

**Figure 3:**
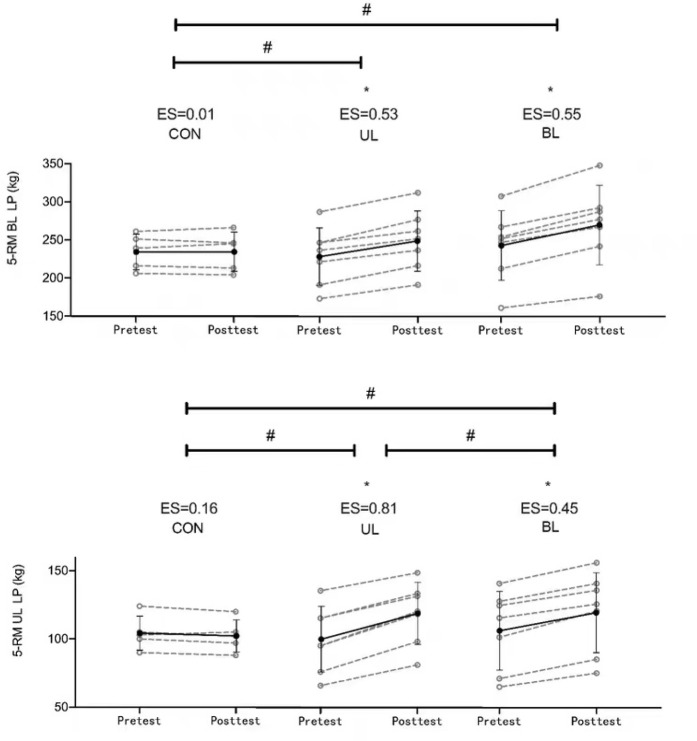
The 5-RM BL LP and UL LP test mean and individual change between pre and post training. *Significant within group difference #Significant between group difference UL LP = unilateral leg press; BL LP = bilateral leg press; CON = control

Of the 26 participants, 19 (seven for UL, seven for BL and five for CON) were included in the final data analysis as four participants were removed due to injury from rugby training and three participants failed to attend over 80% of the training sessions. No significant differences were found for body height (F2,23 = 0.078, *p* = 0.925), body mass (F2,23 = 0.06, *p* = 0.942), age (F2,23 = 0.342, *p* = 0.714) and maturity offset (F2,23 = 0.004, *p* = 0.996) between groups. Other results are summarized in Table 2 and Figure 3.

**Table 2:** Mean and SD of strength, CMJ and sprint tests pre and post training by group.

Test	Group	Pretest	Posttest	Difference (%)	ES
CMJ height (cm)	CON	34.9 ± 5.8	35.8 ± 6.0	2.5	0.15
	UL	37.7 ± 3.0	38.6 ± 4.5	2.3	0.24
	BL	37.8 ± 5.6	38.3 ± 5.7	1.3	0.09
CMJ PP (W)	CON	3227 ± 635	3327 ± 736	3.1	0.15
	UL	3554 ± 210	3665 ± 303	3.1	0.43
	BL	3459 ± 527	3516 ± 562	1.6	0.10
30-m linear sprint (s)	CON	4.75 ± 0.40	4.78 ± 0.37	0.6	0.08
	UL	4.66 ± 0.24	4.71 ± 0.28	1.1	0.19
	BL	4.70 ± 0.34	4.67 ± 0.31	−0.6	0.09

CMJ = countermovement jump; BL = bilateral training group; UL = unilateral training group; CON = control group; ES = effect size; PP = peak power

There was a significant main effect of time for the 5-RM BL LP (F1, 16 = 100.32, *p* ≤ 0.01, $n_{p}^{2}$ = 0.86), and a significant interaction effect (F2, 16 = 23.38, *p* ≤ 0.01, $n_{p}^{2}$ = 0.75). After five weeks of training, both the UL (t6 = −8.556, *p* ≤ 0.01, ES = 0.53) and BL (t6 = −8.773, *p* ≤ 0.01, ES = 0.55) groups experienced significant improvements in the 5-RM BL LP, while no improvement was seen in the CON (t4 = −0.09, *p* = 0.933, ES = 0.01). There was a significant difference in the 5-RM BL LP pre-to-post training change score between groups (F2, 16 = 23.379, *p* ≤ 0.01), with UL and BL groups presenting increases in strength greater than the CON (*p* ≤ 0.01), but no significant difference between the UL and BL LP (*p* = 0.22).

The 5-RM UL LP also showed a significant main effect of time (F_1, 16_ = 115.6, *p* ≤ 0.001, $n_{p}^{2}$= 0.88) and a significant interaction effect (F_2, 16_ = 39.62, *p* ≤ 0.001, $n_{p}^{2}$= 0.83). Following training, BL (t_6_ = −10.74, *p* ≤ 0.01, ES = 0.45) and UL (t_6_ = −9.27, *p* ≤ 0.01, ES=0.81) groups experienced significant improvements in the 5-RM UL LP, while no improvement was seen in the CON (t4 =1.570, *p* = 0.19, ES = 0.01). There was a significant main effect between groups (F2, 16 = 50.38, *p* ≤ 0.01) for the preto-post training change score. Both UL and BL groups had greater strength increases than the CON (*p* ≤ 0.01) and the UL group had significantly greater strength increases than the BL group (*p* = 0.02).

There was no significant effect of time for the 30-m linear sprint (F2, 13 = 0.002, *p* = 0.968, $n_{p}^{2}$≤ 0.01), CMJ height (F2, 13 = 4.606, *p* = 0.051, $n_{p}^{2}$= 0.262) or CMJ PP (F1,13 = 0.959, *p* = 0.345, $n_{p}^{2}$= 0.069), nor was there a significant interaction effect of time and group for the 30-m linear sprint (F2, 13 =1.251, *p* = 0.319, 0.720^,^ $n_{p}^{2}$= 0.161), CMJ height (F2, 13 = 0.336, *p* = $n_{p}^{2}$= 0.049) or CMJ PP (F2, 13 = 0.385, *p* = 0.385, $n_{p}^{2}$= 0.136).

## Discussion

This study is the first to compare the effects of UL and BL LP training on lower body muscle strength, linear sprinting and vertical jumping performance in adolescent rugby players. The results indicate that UL LP training is as effective as BL LP training in improving BL strength and is more effective in improving UL strength. However, there was no significant effect of either training modality on vertical jumping or linear sprinting performance.

Both the UL and BL groups demonstrated significant improvements in the 5-RM BL LP while the CON did not. Although the increase in the 5-RM BL LP for the BL group (10.9%; ES = 0.55) was larger than in the UL group (8.9%; ES = 0.53), there was no significant difference between the two training groups. The increase in strength in the BL group was consistent with other studies (Glowacki et al., 2004; Rossi et al., 2018; Wirth et al., 2016) in which significant increases were found after LP training. Glowacki et al. (2004) reported that 12 weeks of resistance exercise including the LP, leg curl and standing calf raise, resulted in a 19.9% improvement in the 1-RM LP test in untrained male adults. Even greater improvement was seen by Rossi et al. (2018) who found that after 10 weeks of LP training, the 1-RM LP strength improved by 35.2% in a male university student population. Wirth et al. (2016) showed significantly improved 1-RM LP strength (27.6%) after eight weeks of LP training in university students. It is not surprising that the current study demonstrated less improvement because the abovementioned studies used longer periods of training and employed untrained participants.

Our study is the first to report on the effect of UL LP training on BL strength in adolescent rugby players. A similar improvement in the 5-RM BL LP between BL and UL groups indicates that UL training can be as effective as BL training in terms of improving BL strength. It has been suggested that there is comparable electromyography (EMG) in the UL and BL LP when relative intensities are matched, as the movements were performed in a fixed trajectory and with a similar movement pattern (MacDonald et al., 2014). Therefore, the similar 5-RM BL LP strength adaptation could be attributed to comparable neuromuscular activations with the same load performed unilaterally and bilaterally.

Both training groups showed significant improvements in 5-RM UL LP strength, with the improvement significantly larger in the UL group (20.2%; d = 0.81) than in the BL group (12.4%; d = 0.45). No other studies have examined the effectiveness of the BL and UL LP in UL strength measurements. However, some studies that have compared UL and BL squat exercise on UL and BL strength measurements reported no difference between UL and BL training in healthy adults and rugby players (Jones et al., 2012; Speirs et al., 2016). It seems that in our population with the use of the BL/UL LP exercise, UL training could be more effective than BL training in improving UL strength. A possible explanation for this might be that UL training offered more training specificity to the UL tests (Morton et al., 2019). Additionally, because of the existence of the bilateral deficit phenomenon (total amount of force produced during BL contraction is less than the sum of two UL contractions), the UL group received greater training loads compared with the BL group and this could be another mechanism for the greater UL strength improvement in the UL training group (Costa et al., 2015; Jones et al., 2012).

No statistically significant difference was found for the CMJ in the UL, BL or CON groups. However, there was a small to medium effect size in the CMJ test variables (Table 1). These results are in line with authors who reported no statistically significant change for the CMJ in the LP group, after eight weeks of training, in a young adult population (Toumi et al., 2004). Interestingly, in that study, with the same protocol, a squat group showed significantly improved (12.0%) CMJ performance. A similar result was seen by Toumi et al. (2004) with no significant difference in CMJ performance compared with the pre-test value after six weeks of LP training. Blanchard et al. (2015) also found no meaningful improvement in the vertical jump after 8 weeks of LP training in an adolescent population. It is believed that free weight exercise is superior to machine-based exercise for promoting transfer to sport-specific and functional skills (Stone et al., 2002). This is probably because of mechanical specificity, as free weight exercises are closer to the movement pattern, force application and velocity of the movements when performing functional tasks (Stone et al., 2002). Furthermore, free weight squat exercises induce more muscle activation in the lower limbs than Smith the machine squat (Schwanbeck et al., 2009). In addition, free weight exercise results in a greater acute hormonal response than machine weight exercise (Shaner et al., 2014). These results differ from Rossi et al. (2018) who indicated a significantly improved CMJ (3.3%) after 10 weeks of LP training, which is greater than the change in our study. However, it is worth noting that other additional lower extremity RT or endurance training may have been confounding factors that impact the reliability of the study. Wirth et al. (2016) noted that it was preferable to use the squat rather than the LP to improve jump performance, and as a machine-based exercise, the LP itself was questioned regarding the limited transfer effect compared with the free weight exercise like the squat.

Unlike BL LP exercise, there is not yet any published research focused on the effect of the UL LP on the CMJ. Wawrzyniak et al. (1996) found that after six weeks (three sessions per week) of UL LP training in a group of untrained young adults, there was no statistically significant difference in single-leg hop distance compared with the CON, which is in line with our findings.

No significant pre-post difference in the 30-m linear sprint test was also found for all three groups. This finding is also consistent with Blanchard et al. (2015), who did not find significant changes in the 40-yard (36.6 m) sprint in adolescent boys after eight weeks of LP training. No study thus far has investigated the effect of the UL LP on sprint performance. Because CMJ power is strongly correlated with sprint performance (r = -0.68) in team sport athletes, it is not surprising that sprint performance did not show significant improvement either (Sleivert et al., 2004). The lack of a main effect for 30-m linear sprinting tests in this study may be attributed to the short duration of the training period. Five weeks of training may be too short for adolescent players to transfer strength gain to an improvement in the 30-m linear sprint as well as the CMJ. Another explanation for the lack of improvement in vertical jumping and sprinting performance could be the lag-time effect. The term ‘lag time’ refers to the delay between increased muscle strength and the ability to actualize increased strength into the improved performance like sprinting and jumping (Nimphius, 2010). The length of lag time may vary for different exercises used for training and it could last for as long as one month (Stone, 1987). Furthermore, the higher level of sprint acceleration performance is more related to the horizontally-oriented ground reaction force vector (Bezodis et al., 2017). It is therefore not surprising to find no significant effects on the 30-m linear sprint as the LP is performed in a vertical direction relative to the body position.

The major limitation of our study was the relatively small sample size, partially limiting the generalizability and interpretation of our findings. Our study was also limited by the short period of training and only one resistance exercise performed in each group. Combined these may not be sufficient to elicit training effects, at least for athletic performance outcomes such as vertical jumping and linear sprinting.

In conclusion, 5 weeks of UL or BL LP training significantly improved BL lower body strength of adolescent rugby players to a similar extent, and UL LP training was more effective for improving UL lower body strength. Neither UL nor BL LP training significantly improved vertical jumping or linear sprinting performance. The results of this study indicate that 5 weeks of UL or BL LP training performed twice per week using five sets and relative intensities ranging from 70 to 90% can significantly improve BL strength to a similar extent in adolescent rugby players. However, UL LP training may be preferable to the BL LP in improving UL strength. If practitioners aim to improve BL lower body strength of adolescent rugby players, they may consider incorporating the UL and/or the BL LP twice per week using five sets and relative intensities ranging from 70 to 90% for at least 5 weeks. If the aim is to improve UL strength, the UL LP should be used with the same training variables as above. When the target is to improve vertical jumping and linear sprinting, practitioners should probably try to extend the training period or use alternative approaches

## References

[ref1] Baker, D. G., & Newton, R. U. (2008). Comparison of lower body strength, power, acceleration, speed, agility, and sprint momentum to describe and compare playing rank among professional Rugby league players. Journal of Strength and Conditioning Research, 22(1), 153–158. 10.1519/jsc.0b013e31815f9519 18296969

[ref2] Bezodis, N. E., North, J. S., & Razavet, J. L. (2016). Alterations to the orientation of the ground reaction force vector affect sprint acceleration performance in team sports athletes. Journal of Sports Sciences, 35(18), 1817–1824. 10.1080/02640414.2016.1239024 27700312

[ref3] Blanchard, J., & DeBeliso, M. (2015). Effects of the trap bar dead lift and leg press on early adolescent males leg strength, vertical jump and sprint performance. Medicine & Science in Sports & Exercise, 47(5S), 929. 10.1249/01.mss.0000479251.73138.f1

[ref4] Blimkie C, & Sale, D. (1998). *Pediatric Anaerobic Performance*. Champaign, Illinois: Human Kinetics.

[ref5] Bobbert, M. F., de Graaf, W. W., Jonk, J. N., & Casius, L. J. (2006). Explanation of the bilateral deficit in human vertical squat jumping. Journal of Applied Physiology, 100(2), 493–499. 10.1152/japplphysiol.00637.2005 16239616

[ref6] Chelly, M. S., Fathloun, M., Cherif, N., Amar, M. B., Tabka, Z., & Van Praagh, E. (2009). Effects of a back squat training program on leg power, Jump, and sprint performances in junior soccer players. Journal of Strength and Conditioning Research, 23(8), 2241–2249. 10.1519/jsc.0b013e3181b86c40 19826302

[ref7] Correa, C., LaRoche, D., Cadore, E., Reischak-Oliveira, A., Bottaro, M., Kruel, L., Tartaruga, M., Radaelli, R., Wilhelm, E., Lacerda, F., Gaya, A., & Pinto, R. (2012). 3 different types of strength training in older women. International Journal of Sports Medicine, 33(12), 962–969. 10.1055/s-0032-1312648 22782384

[ref8] Costa, E., Moreira, A., Cavalcanti, B., Krinski, K., & Aoki, M. (2014). undefined. Biology of Sport, 32(1), 35–40. 10.5604/20831862.1126326 25729148 PMC4314602

[ref9] Dobbin, N., Hunwicks, R., Highton, J., & Twist, C. (2018). A reliable testing battery for assessing physical qualities of elite Academy Rugby league players. Journal of Strength and Conditioning Research, 32(11), 3232–3238. 10.1519/jsc.0000000000002280 29140912

[ref10] Epley, B. (1985). Poundage chart: Boyd Epley workout. Body Enterprises, Lincoln, NE, 86.

[ref11] Faigenbaum, A. D., Kraemer, W. J., Blimkie, C. J., Jeffreys, I., Micheli, L. J., Nitka, M., & Rowland, T. W. (2009). Youth resistance training: Updated position statement paper from the national strength and conditioning association. Journal of Strength and Conditioning Research, 23(Supplement 5), S60–S79. 10.1519/jsc.0b013e31819df407 19620931

[ref12] Faigenbaum, A. D., & Myer, G. D. (2009). Resistance training among young athletes: Safety, efficacy and injury prevention effects. British Journal of Sports Medicine, 44(1), 56–63. 10.1136/bjsm.2009.068098 19945973 PMC3483033

[ref13] Glowacki, S. P., Martin, S. E., Maurer, A., Baek, W., Green, J. S., & Crouse, S. F. (2004). Effects of resistance, endurance, and concurrent exercise on training outcomes in men. Medicine & Science in Sports & Exercise, 2119–2127. 10.1249/01.mss.0000147629.74832.52 15570149

[ref14] Gonzalo-Skok, O., Tous-Fajardo, J., Suarez-Arrones, L., Arjol-Serrano, J. L., Casajús, J. A., & MendezVillanueva, A. (2017). Single-leg power output and between-limbs imbalances in team-sport players: Unilateral versus bilateral combined resistance training. International Journal of Sports Physiology and Performance, 12(1), 106–114. 10.1123/ijspp.2015-0743 27140680

[ref15] Gorostiaga, E. M., Izquierdo, M., Iturralde, P., Ruesta, M., & Ibáñez, J. (1999). Effects of heavy resistance training on maximal and explosive force production, endurance and serum hormones in adolescent handball players. European Journal of Applied Physiology and Occupational Physiology, 80(5), 485–493. 10.1007/s004210050622 10502084

[ref16] Haff GG, & Triplett, NT. (2015). Essentials of strength training and conditioning 4th edition; Champaign: Human Kinetics.

[ref17] Hopkins, W. G. (2004). How to interpret changes in an athletic performance test. Sport Science, 8, 1–7.

[ref18] Jones, M. T., Ambegaonkar, J. P., Nindl, B. C., Smith, J. A., & Headley, S. A. (2012). Effects of unilateral and bilateral lower-body heavy resistance exercise on muscle activity and testosterone responses. Journal of Strength and Conditioning Research, 26(4), 1094–1100. 10.1519/jsc.0b013e318248ab3b 22222320

[ref19] MacDonald, M., Losier, D., Chester, V. L., & Kuruganti, U. (2014). Comparison of bilateral and unilateral contractions between swimmers and nonathletes during leg press and hand grip exercises. Applied Physiology, Nutrition, and Metabolism, 39(11), 1245–1249. 10.1139/apnm-2014-0040 25140863

[ref20] McCurdy, K. W., Langford, G. A., Doscher, M. W., Wiley, L. P., & Mallard, K. G. (2005). The effects of short-term unilateral and bilateral lower-body resistance training on measures of strength and power. Journal of Strength and Conditioning Research, 19(1), 9. 10.1519/14173.1 15705051

[ref21] Mirwald, R. L., Baxter-Jones, A. D., Bailey, D. A., & Beunen, G. P. (2002). An assessment of maturity from anthropometric measurements. Medicine Science in Sports & Exercise, 34(4), 689–694. 10.1249/00005768-200204000-00020 11932580

[ref22] Morton, R. W., Colenso-Semple, L., & Phillips, S. M. (2019). Training for strength and hypertrophy: An evidence-based approach. Current Opinion in Physiology, 11, 149–150. 10.1016/j.cophys.2019.08.002

[ref23] Myers, A. M., Beam, N. W., & Fakhoury, J. D. (2017). Resistance training for children and adolescents. Translational Pediatrics, 6(3), 137–143. 10.21037/tp.2017.04.01 28795003 PMC5532191

[ref24] Nimphius, S. (2010). Lag time: The effect of a two week cessation from resistance training on force, velocity and power in elite softball players. Journal of Strength and Conditioning Research, 24, 1. 10.1097/01.jsc.0000367186.47762.66

[ref25] Pamukoff, D. N. , Haakonssen, E. C., Zaccaria, J. A., Madigan, M. L., Miller, M. E., & Marsh, A. P. (2014). The effects of strength and power training on single-step balance recovery in older adults: a preliminary study. Clinical Intervention in Aging, 9, 697–704. doi:10.2147/cia.S59310 PMC4000185 24790422

[ref26] Quarrie, K. L., Handcock, P., Waller, A. E., Chalmers, D. J., Toomey, M. J., & Wilson, B. D. (1995). The New Zealand Rugby injury and performance project. III. Anthropometric and physical performance characteristics of players. British Journal of Sports Medicine, 29(4), 263–270. 10.1136/bjsm.29.4.263 8808542 PMC1332239

[ref27] Romero-Franco, N., Jiménez-Reyes, P., Castaño-Zambudio, A., Capelo-Ramírez, F., Rodríguez-Juan, J. J., González-Hernández, J., Toscano-Bendala, F. J., Cuadrado-Peñafiel, V., & Balsalobre-Fernández, C. (2016). Sprint performance and mechanical outputs computed with an iPhone app: Comparison with existing reference methods. European Journal of Sport Science, 17(4), 386–392. 10.1080/17461391.2016.1249031 27806673

[ref28] Rossi, F. E., Schoenfeld, B. J., Ocetnik, S., Young, J., Vigotsky, A., Contreras, B., Krieger, J. W., Miller, M. G., & Cholewa, J. (2018). Strength, body composition, and functional outcomes in the squat versus leg press exercises. Journal of Sports Medicine and Physical Fitness, 58(3). 10.23736/s0022-4707.16.06698-6 27735888

[ref29] Schwanbeck, S., Chilibeck, P. D., & Binsted, G. (2009). A comparison of free weight squat to Smith machine squat using electromyography. Journal of Strength and Conditioning Research, 23(9), 2588–2591. 10.1519/jsc.0b013e3181b1b181 19855308

[ref30] Shaner, A. A., Vingren, J. L., Hatfield, D. L., Budnar, R. G., Duplanty, A. A., & Hill, D. W. (2014). The acute hormonal response to free weight and machine weight resistance exercise. Journal of Strength and Conditioning Research, 28(4), 1032–1040. 10.1519/jsc.0000000000000317 24276305

[ref31] Sleivert, G., & Taingahue, M. (2004). The relationship between maximal Jump-squat power and sprint acceleration in athletes. European Journal of Applied Physiology, 91(1), 46–52. 10.1007/s00421-003-0941-0 14508691

[ref32] Smart, D. J., & Gill, N. D. (2013). Effects of an off-season conditioning program on the physical characteristics of adolescent Rugby union players. Journal of Strength and Conditioning Research, 27(3), 708–717. 10.1519/jsc.0b013e31825d99b0 22652917

[ref33] Speirs, D. E., Bennett, M. A., Finn, C. V., & Turner, A. P. (2016). Unilateral vs. bilateral squat training for strength, sprints, and agility in Academy Rugby players. Journal of Strength and Conditioning Research, 30(2), 386–392. 10.1519/jsc.0000000000001096 26200193

[ref34] Stone M., Plisk, S., & Collins, D. (2002). Training principles: evaluation of modes and methods of resistance training--a coaching perspective. Sports biomechanics, 1(1), 79–103. doi:10.1080/14763140208522788 14658137

[ref35] Stone MH. (1987). *Weight training: A Scientific Approach*. Minneapolis: Burgess international.

[ref36] Toumi, H., Best, T. M., Martin, A., & Poumarat, G. (2004). Muscle plasticity after weight and combined (Weight + Jump) training. Medicine & Science in Sports & Exercise, 36(9), 1580–1588. 10.1249/01.mss.0000139896.73157.21 15354041

[ref37] Wawrzyniak, J.R., Tracy, J.E., Catizone, P.V., & Storrow, R.R. (1996). Effect of closed chain exercise on quadriceps femoris peak torque and functional performance. Journal of Athletic Training, 31(4), 335–340. 16558420 PMC1318918

[ref38] Wirth, K., Hartmann, H., Sander, A., Mickel, C., Szilvas, E., & Keiner, M. (2016). The impact of back squat and leg-press exercises on maximal strength and speed-strength parameters. Journal of Strength and Conditioning Research, 30(5), 1205–1212. 10.1519/jsc.0000000000001228 26439782

